# Population pharmacokinetic, pharmacodynamic and efficacy modeling of SB12 (proposed eculizumab biosimilar) and reference eculizumab

**DOI:** 10.1007/s00228-024-03703-8

**Published:** 2024-05-30

**Authors:** Hyuna Lee, Jihye Park, Hyerin Jang, So Jin Lee, Jungryul Kim

**Affiliations:** 1 Samsung Bioepis, Co., Ltd, Incheon, Republic of Korea; 2 AIMS BioScience, Seoul, Republic of Korea; 3 Samsung Medical Center, Seoul, Republic of Korea

**Keywords:** Eculizumab, SB12, Biosimilar, Paroxysmal nocturnal hemoglobinuria, Population modeling

## Abstract

**Purpose:**

To describe, compare similarity of pharmacokinetic (PK), pharmacodynamic (PD) and efficacy of SB12 and reference eculizumab (ECU) and find clinically significant covariate relationships.

**Methods:**

The PK, PD (terminal complement activity) and efficacy (LDH) data of SB12 and ECU were obtained from 289 subjects from phase I and phase III studies. One- and two-compartment PK models with first-order elimination were evaluated for SB12 and ECU. For PD and efficacy, both direct and indirect models were tested. The impact of covariates on PK, PD and efficacy parameters was assessed. Relationship between PK/PD and PD/efficacy was characterized. This modeling was performed using NONMEM version 7.4 (Icon Development Solutions, Ellicott City, MD, USA).

**Results:**

The two-compartment model adequately described the PK of SB12 and ECU, and the subject’s weight was chosen as a clinically significant covariate affecting drugs’ clearance and central volume of distribution. Treatment group was not a significant covariate affecting clearance. The direct response model using inhibitory sigmoid *E*_max_ and sigmoid *E*_max_ relationship well described the PK/PD relationship and PD/efficacy relationship of SB12 and ECU, respectively. Through this modeling, the relationships between PK, PD and efficacy were characterized. There were no differences in PK, PD and efficacy parameters between SB12 and ECU in pooled populations of healthy subjects and paroxysmal nocturnal haemoglobinuria (PNH) patients.

**Conclusion:**

The population modeling showed PK, PD and efficacy similarities between SB12 and ECU in pooled population of healthy subjects and PNH patients, supporting the totality of evidence on biosimilarity for SB12.

**Supplementary Information:**

The online version contains supplementary material available at 10.1007/s00228-024-03703-8

## Introduction

Paroxysmal nocturnal haemoglobinuria (PNH) is a rare haematological disorder that presents clinically with various symptoms, the most prevalent of which are characterized by haemolytic anaemia and pancytopaenia [1]. The incidence of PNH is estimated at 1–1.5 cases per million individuals worldwide, but it is higher in certain countries (e.g. Japan, Korea and China) in Asia than in Western countries [1]. The most predominantly occurred age range was 30 to 40 years, and the incidence in children is rare [1, 2]. PNH is caused by the development of a genetic mutation of the X-linked gene phosphatidylinositol glycan class A (PIGA), which produces a deficiency in the glycosylphosphatidylinositol (GPI)-anchored terminal inhibitors (e.g. CD55, CD59), in haematopoietic stem cells [3, 4]. CD55 and CD59, specific proteins responsible for the regulation of complement activity, are thereby prevented from attaching to PNH-affected cells. The resultant loss of complement inhibition causes chronic complement-mediated haemolysis of PNH cells. Therefore, the current main therapy for PNH includes drugs to block alternative complement pathways such as eculizumab [5–8].

Eculizumab, a humanized monoclonal immunoglobulin G2/4 (IgG2/4) kappa antibody, was the first approved therapy for patients with PNH in 2007 [9, 10]. It binds to the terminal complement protein C5, which acts as a late stage in the complement cascade, inhibits the complement pathway by preventing the cleavage of C5 to C5a and C5b, and thereby reduces haemolysis and thrombotic risk [11, 12]. Therefore, eculizumab recompenses for CD59 deficiency in patients with PNH [1]. Eculizumab has been safely administered to over 1000 patients with PNH worldwide over 14 years as of 2014 [13]. It can resolve intravascular haemolysis, reduce thrombosis rate, improve or stabilize renal function and improve pulmonary pressure and survival [14–22], and it is currently used for the treatment of PNH, atypical haemolytic uremic syndrome, generalized myasthenia gravis and neuromyelitis optica spectrum disorders.

SB12 was developed as a European Union (EU)–approved biosimilar product of reference eculizumab (ECU) (Soliris, Alexion Pharmaceuticals, Inc., Boston, MA, USA). The pharmaceutical form, composition, strength of the product and route of administration of SB12 are identical to that of the reference products sourced from the EU (EU-ECU) and the United States (US-ECU) [23]. The phase I single-dose study was conducted to compare the pharmacokinetics (PK), pharmacodynamics (PD), safety, tolerability and immunogenicity of SB12, EU-ECU and US-ECU [24]. For the comparisons of SB12 to each of the two ECUs and the comparison of EU-ECU to US-ECU, the 90% confidence intervals (CIs) for the ratio of geometric least squared means (LSMeans) of primary PK parameters were within the predefined bioequivalence margins of 80.00–125.00%. The PD, safety, tolerability and immunogenicity profiles were comparable in healthy subjects. In addition to the PK similarity study in healthy subjects, the PK and PD of SB12 and ECU have been characterized in a comparative clinical study in patients with PNH [25]. The phase III cross-over multiple-dose study was performed to compare the efficacy, safety, PK, PD and immunogenicity of SB12 and ECU in patients with PNH. This study demonstrated equivalent clinical efficacy of SB12 and ECU, and PK, PD, safety and immunogenicity were comparable between SB12 and ECU. The eculizumab dose depends on the indication and weight of the patient (dose adjustment for patients with a body weight < 40 kg), and the serum eculizumab concentration depends on these factors. The population PKs of eculizumab in PNH patients have been described using a one-compartment model [20], and PK parameters of multiple-dose studies conducted in three patients groups with rheumatoid arthritis (RA), idiopathic membranous glomerulopathy (IMG) and PNH were described using a two-compartment model. At therapeutic dose, eculizumab shows linear PK, indicating saturation of the drug target [10, 26]. In previous study, the relationship between total complement activity (CH50) and lactate dehydrogenase (LDH) in PNH patients was assessed using a linear mixed model with intercept and slope for therapy duration. Complete blockade of complement activity was significantly correlated with lower LDH levels [27].

To our knowledge, no population PK/PD/efficacy model for ECU using pooled data of healthy subjects and PNH patients has been reported. The objectives of the population PK/PD/efficacy modeling were to characterize the population PK, PD and efficacy of SB12 and ECU and to evaluate the impact of covariates, including treatment (i.e. SB12 versus ECU), on the PK, PD and efficacy parameters.

## Materials and methods

### Study design and subjects

Data from the phase I clinical trial in healthy subjects and the phase III clinical trial in patients with PNH were pooled to conduct this population PK/PD/efficacy analysis (Table 1). A total of 240 healthy subjects and 50 patients with PNH were enrolled in phase I and phase III clinical trials, respectively, and these subjects’ data were used in this analysis. A randomized, double-blinded, three-arm, single-dose phase I study was conducted in healthy subjects, and a phase III randomized, double-blinded, multicentre, cross-over study was conducted in patients with PNH. The final protocol and all amendments were approved by the responsible local Independent Ethics Committee (IEC) or Institutional Review Board (IRB). These studies were conducted in accordance with the International Council for Harmonisation and Good Clinical Practice guidelines and the Declaration of Helsinki (2013). A written informed consent form was signed by each subject before the study enrolment and approved by the IEC or IRB prior to use.

**Table 1:** Summary of clinical studies used in this population pharmacokinetic/pharmacodynamic/efficacy modeling

**Study**	**Dosing regimen**	**Treatment**	**Number of subjects**	**Scheduled sampling time points**
Phase I (healthy subjects)	300 mg single dose	SB12 EU-ECU US-ECU	240 (80 subjects per treatment group)	PK: 0 (pre-dose), 0.58 (35 min: immediately after the end of infusion), 4, 8, 12, 24, 48, 96, 168, 240, 336, 504, 672, 840, 1008, 1176, 1344 and 1512 h after the start of infusion PD: 0, 0.58, 4, 24, 48, 96, 168, 240, 336 and 1512 h after the start of infusion
Phase III (PNH patients)	Induction: 600 mg every week for the first 4 weeks Maintenance: 900 mg for the fifth week, followed by 900 mg every 2 weeks until week 50	Treatment sequence I: SB12 to ECU Treatment sequence II: ECU to SB12	49^a^ (Treatment sequence I: 24 patients; Treatment sequence II: 25 patients)	PK: Pre-dose at Week 0, 2, 4, 6, 10, 14, 18, 22, 26, 28, 30, 32, 36, 40, 44, 48 and 52 PD: Week 0, 2, 4, 6, 10, 14, 26, 28, 30, 32, 36, 40 and 52 Efficacy: Week 0, 1, 2, 3, 4, 6, 8, 10, 12, 14, 16, 18, 20, 22, 24, 26, 28, 30, 32, 34, 36, 38, 40, 42, 44, 46, 48, 50 and 52

*ECU* reference eculizumab, *EU-ECU* EU-sourced Soliris, *PD* pharmacodynamics, *PK* pharmacokinetics, *PNH* paroxysmal nocturnal haemoglobinuria, *US-ECU* US-sourced Soliris

^a^One patient assigned to treatment sequence I did not receive study treatment and was excluded from the analysis. During the study, there was an unplanned IP switch due to a comparator shortage in eight patients in treatment sequence I at period 2

For the assessment of PK, PD and efficacy for SB12 and ECU, serum samples were collected at prespecified time points in each of the studies (Table 1). PK, PD and efficacy samples were analysed by qualified laboratories. The serum concentration of eculizumab for PK was measured using the electrochemiluminescent format with acid dissociation specific for the detection and quantification of eculizumab [24], and the lower limit of quantification (LLOQ) and upper limit of quantification (ULOQ) were 0.8 μg/mL and 12.5 μg/mL, respectively. The amount of neoepitope generated by the formation of the terminal complement complex for PD was measured using the validated enzyme-linked immunosorbent assay, and the LLOQ and ULOQ were 10% and 125%, respectively. The efficacy endpoint, LDH, was one of disease-related laboratory parameters, and blood samples for this parameter were analysed by a spectrophotometry-based analysis in the central laboratory.

### Data analysis

The population PK/PD/efficacy model was developed using non-linear mixed effect modeling software, NONMEM (version 7.4, Icon Development Solutions, Ellicott City, MD, USA) with a first-order conditional estimation method with interaction (FOCE-INTER). Excel 2010, R (version 4.0.3, R Foundation, Vienna, Austria) and Xpose4 (version 4.7.2; Department of Pharmaceutical Biosciences, Uppsala University) were used for dataset construction, data presentation, construction of plots and graphical exploration.

### Model development

#### Overall analysis strategies

Initially, the PK and PD models were developed using a healthy subject study to find significant covariates affecting PK and PD parameters and assess potential differences between the treatment groups (SB12, EU-ECU and US-ECU) in healthy subjects. Thereafter, the PK and PD models were developed using pooled data from both healthy subjects and PNH patients. Sequentially, efficacy model was established using patients’ data linking previously built PK/PD model for patients to establish PK/PD/efficacy model. From pooled data analysis, the potential difference in PK and PD parameters between healthy subjects and PNH patients was assessed first, followed by a covariate search. The potential difference between the treatment groups was assessed for each model.

#### Base model

The PK model was initially developed using data from the phase I study, and this model served as a backbone for the subsequent PK model incorporating pooled data from phase III. Developing the base model was necessary using serial PK and PD samples from healthy subjects, as only trough serum concentration levels were available from PNH patients. Subsequently, the PK/PD and PK/PD/efficacy models were developed using pooled data from both phase I and phase III studies and patients, respectively. The serum concentration values determined before the first dosing and below limit of quantification were considered missing. The percentage of LLOQ observations in serum concentrations of eculizumab for PK was 14.1%. All available serum concentrations, terminal complement activity and LDH data of the subjects were included in the dataset. If the baseline value of LDH was missing, the value collected at screening was used instead, which is the closest to samples from visit 1 (day 1). One- and two-compartment models with first-order elimination were tested for PK model of SB12 and ECU. For the PD and efficacy models, both direct and indirect models were tested for the terminal complement activity-time and LDH-time profiles of SB12 and ECU. The inter-individual variability (i.e. ETA) of each parameter was applied exponentially. Additive, proportional or combined error models were evaluated for residual variability. When linking two models such as PK to PD and PK/PD to efficacy, the individual population prediction (IPP) method, which is one of the sequential methods to link PK to PD, allows predicting subsequently linked model parameters of individuals based on the individual predictions of a preceding model reflecting ETA [28].

#### Covariate model

The covariate model was built in a stepwise fashion with forward inclusion (*p* value = 0.05) and backward elimination (*p* value = 0.01). The list of covariates tested included age, weight, height, BMI, sex, race, subject group and period, sequence and phase of study. Additionally, clinical laboratory variable related to the symptoms of the disease and potential complications of the disease, such as laboratory variables for renal and hepatic functions, were also screened. The potential covariates were identified based on generalized additive modeling (GAM) implemented in Xpose4 and inspection of ETA-covariate and covariate-covariate plots. The contribution of the remaining covariates was evaluated by eliminating each covariate from the model one at a time. Continuous variables were transformed using a power model centred on the median value for the corresponding variable. Categorical variables were incorporated and typical values were separately estimated for each category.

### Model evaluation

Model validation was performed using prediction-corrected Visual Predictive Check (pcVPC) and the bootstrap-resampling method. In the pcVPC step, the final parameter estimates were used to simulate 1000 data points for each observation based on covariates, actual PK, PD, efficacy sampling times and dosing histories recorded in the original dataset. The prediction-corrected observed data points and 50th percentile curves overlaid with prediction-corrected simulated 5th, 50th and 95th percentile curves of 1000 simulated datasets were visually inspected. For each predicted percentiles, 95% confidence intervals were overlaid. The final population PK/PD/efficacy model was evaluated using 1000 bootstrap replicates. The bootstrap estimates were used to obtain 95% CIs for all model parameters including those for the covariate estimates.

## Results

### Subject characteristics

Subject characteristics are summarized in Table 2. The model using phase I study data consisted of 240 healthy subjects (80 subjects per treatment group, a total of 230 males and 10 females). The model using pooled data including phase III study consisted of 49 PNH patients (22 males and 27 females). Dense sampling resulted in a total of 4136 quantifiable serum concentrations, 2900 terminal complement activity levels from 289 subjects and 1350 LDH levels from 49 patients with PNH. A notable difference in weight and height was observed between the subjects from phase I and phase III studies, which may be influenced by a higher percentage of female and Asian race in the phase III study. Clinical laboratory variables were also included in the dataset in which patients showed significantly higher baselines of aspartate aminotransferase, bilirubin and LDH than healthy subjects, consistent with known clinical characteristics of PNH patients related to clinical manifestations and complications of PNH [29–32].

**Table 2:** Summary of subject characteristics

**Characteristic**	**Phase I study** ***N*= 240**	**Phase III study** ***N*= 49**
Age (years)	40 (19–55)	36 (18–79)
Height (cm)	181.0 (162–198)	164.9 (146–190)
Weight (kg)	82.40 (70.0–94.3)	63.00 (43.0–111.0)
BMI (kg/m^2^)	25.50 (20.0–29.9)	23.00 (16.8–37.5)
**Gender, *n* (%)**		
Male	230 (95.8)	22 (44.9)
Female	10 (4.2)	27 (55.1)
**Race, *n* (%)**		
White	230 (95.8)	18 (36.7)
Black or African American	1 (0.4)	0 (0.0)
Asian	1 (0.4)	26 (53.1)
American Indian or Alaska Native	1 (0.4)	0 (0.0)
Native Hawaiian or Other Pacific Islander	0 (0.0)	5 (10.2)
Other	7 (2.9)	18 (36.7)

All continuous data are presented as median (minimum–maximum)

*BMI* body mass index, *N* number of subjects included in PK/PD/efficacy analysis of each clinical study, *n* number of subjects within the category

### Population PK/PD/efficacy analysis

#### PK/PD model of healthy subjects

**Fig. 1:**
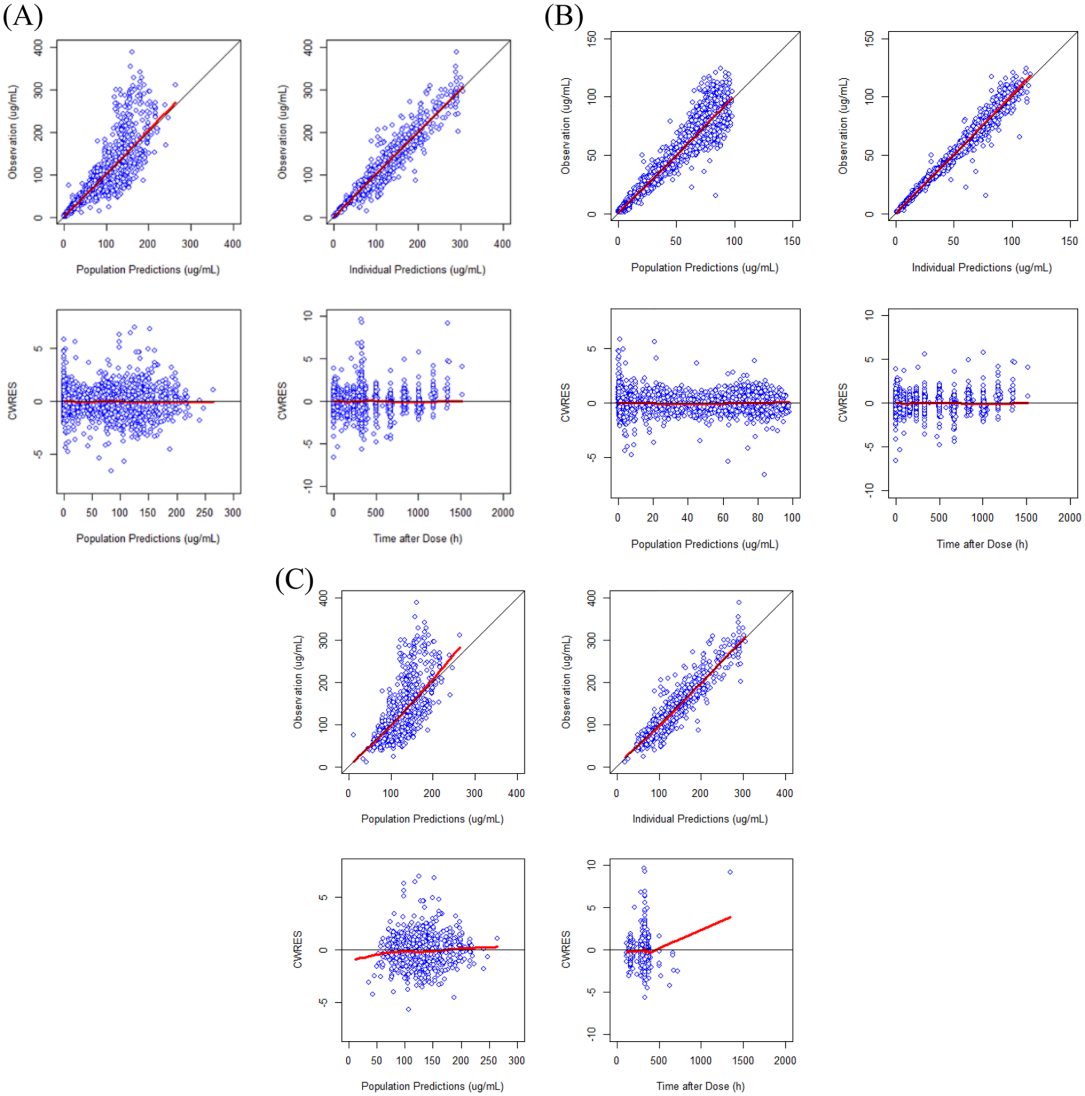
Basic goodness-of-fit plots of the final pharmacokinetic model for SB12 and reference eculizumab of **A** all subjects, **B** healthy subjects, and **C** paroxysmal nocturnal haemoglobinuria patients. CWRES, conditional weighted residuals

**Fig. 2:**
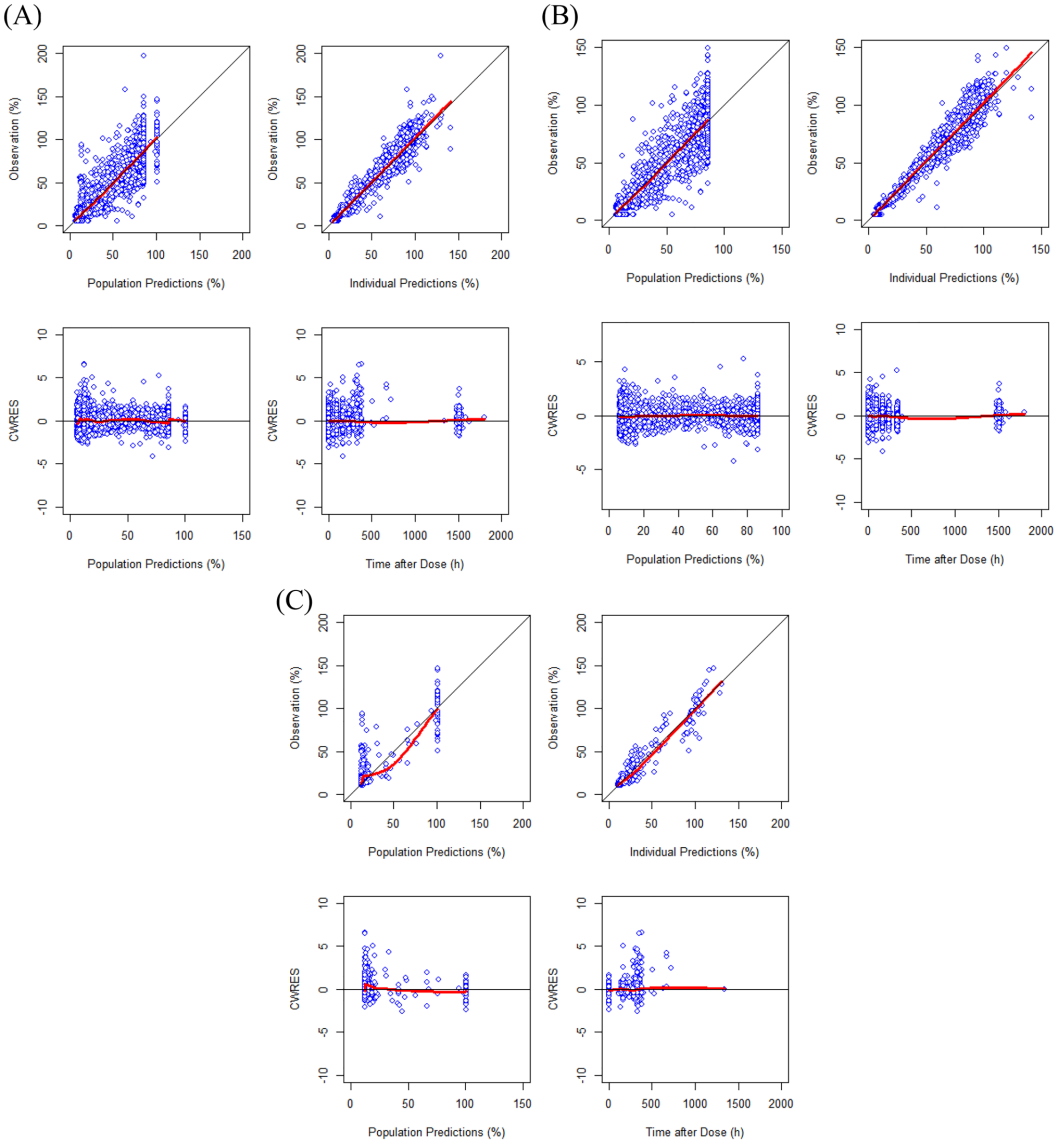
Basic goodness-of-fit plots of the final pharmacodynamic model for terminal complement activity for SB12 and reference eculizumab of **A** all subjects, **B** healthy subjects and **C** Paroxysmal nocturnal haemoglobinuria patients. CWRES, conditional weighted residuals

The population PKs of SB12 and ECU in healthy subjects were best described by a two-compartment model dividing the body into central and peripheral compartment with first-order elimination with a proportional error term for residual variability and ETA on clearance (CL), volume of central compartment (*V*_*c*_) and volume of peripheral compartment (*V*_*p*_) with covariance between *CL* and *V*_*c*_. The population PDs of SB12 and ECU in healthy subjects were well described by a direct response model with an inhibitory sigmoid maximum effect (*E*_max_) relationship between serum concentrations and terminal complement activity. Through covariate analysis in healthy subject, for the PK model, weight was identified as a significant covariate for *CL* and *V*_*c*_ (*p* value < 0.01). The only covariate included in the final PD model was the baseline value of terminal complement activity for serum concentration achieving 50% of the maximum effect of terminal complement activity inhibition (*IC*_50_). The goodness-of-fit (GOF) plots demonstrated that the model predictions were in line with the observed PK and PD values (Figs. 1B and 2B). The VPC (Fig. 4A for PK and Fig. 4C for PD) showed that the final models successfully described the observed data. Final parameter estimates and bootstrap results are summarized in Appendix Tables A1 and A2 for PK and PD models, respectively.

#### PK/PD model of pooled subjects and PK/PD/efficacy model of PNH patients

Similar to healthy subjects, a two-compartment model best described the PKs of SB12 and ECU in pooled data including healthy subjects and PNH patients. PK/PD model from healthy subjects served as a backbone for the pooled data model. A proportional error model was used for residual variability, and ETA was included on *CL*, *V*_*c*_ and *V*_*p*_ with covariance between *CL* and *V*_*c*_. Through covariate analysis, weight was included in the final PK model for *CL* and *V*_*c*_ (*p* value < 0.01). The population PDs using pooled data were well described by a direct response model as in healthy subjects with ETA on baseline terminal complement activity (*E*_0_), maximum effect of terminal complement activity inhibition (*I*_max_) and *IC*_50_. The final PD model incorporated the subject group (healthy subjects vs. PNH patients) as the only covariate for *E*_0_ and *I*_max_.

Furthermore, using patient data alone, an efficacy model was built describing LDH profiles and was linked to the PK/PD model of patient using patient-specific covariate estimates. The relationship between PD and efficacy was best described by the direct sigmoid *E*_max_ model.

**Fig. 3:**
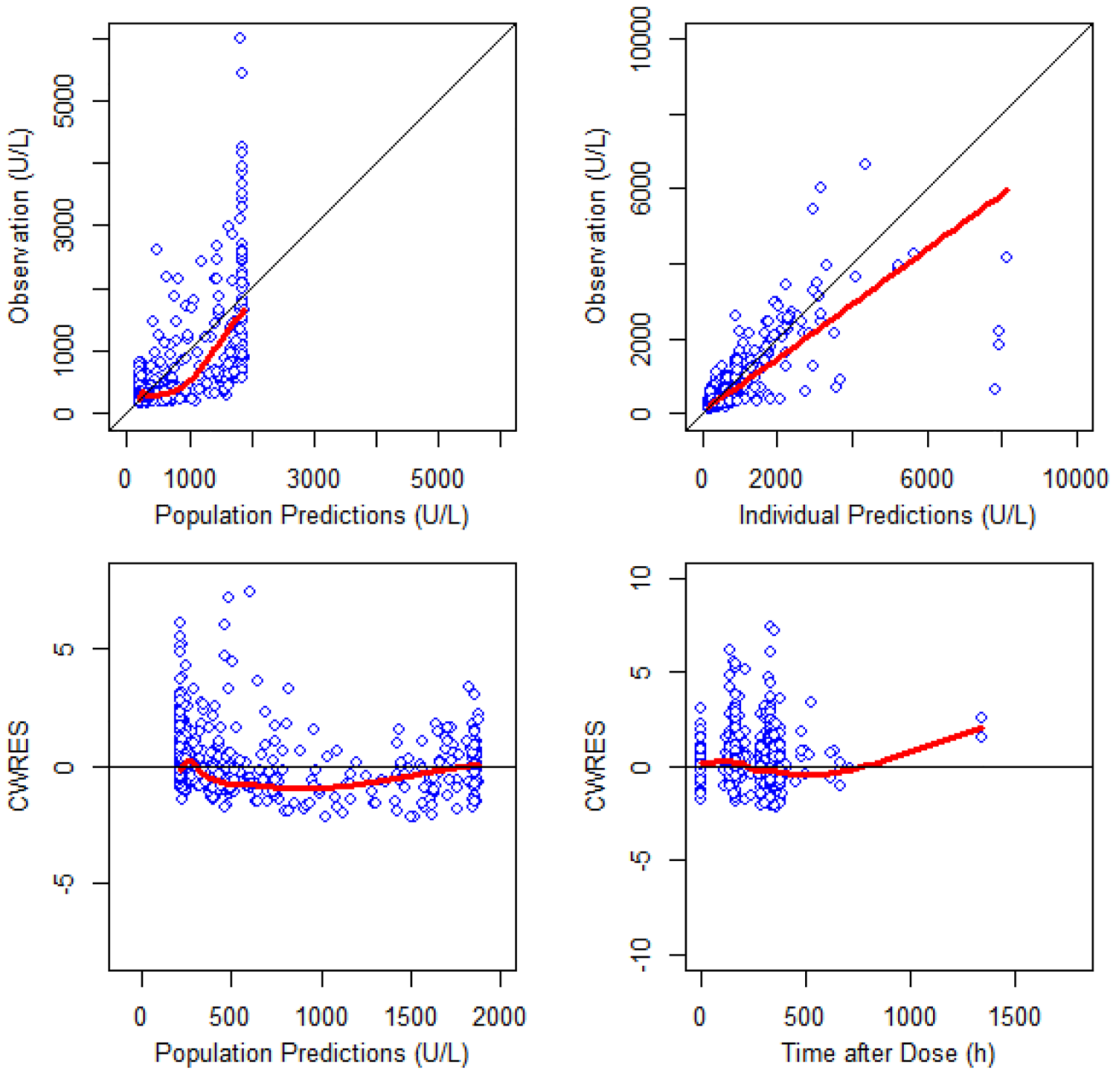
Basic goodness-of-fit plots of the final pharmacokinetic/pharmacodynamic/efficacy model for SB12 and reference eculizumab in paroxysmal nocturnal haemoglobinuria patients, CWRES, conditional weighted residuals

**Fig. 4:**
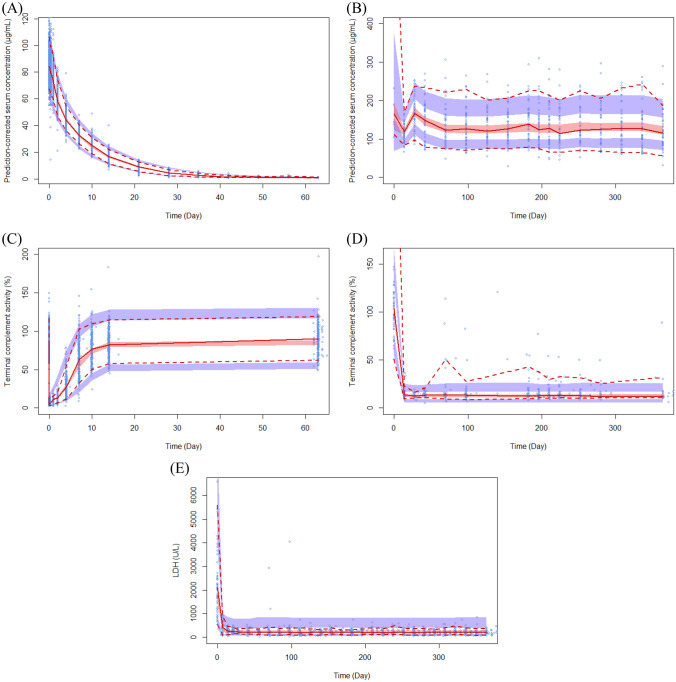
Visual predictive check plots of the final model for SB12 and reference eculizumab. **A**, **B** Final pharmacokinetic model in **A** healthy subjects and **B** paroxysmal nocturnal haemoglobinuria patients. **C**, **D** Final pharmacodynamic model in **C** healthy subjects and **D** paroxysmal nocturnal haemoglobinuria. **E** Final pharmacokinetic/pharmacodynamic/efficacy model in paroxysmal nocturnal haemoglobinuria patients. The solid red line represents the median observed value (prediction-corrected), and the semi-transparent red field represents a simulation-based 95% confidence intervals for the median. The observed 5% and 95% percentiles are presented with dashed red line, and 95% confidence intervals for each model predicted percentiles are shown as semi-transparent blue field

The GOF plots for each PK, PD and PK/PD/efficacy model (Figs. 1, 2 and 3) showed that the final models well described the observed values of pooled data in each subject group. VPC plots (Fig. 4) showed that the model described the observed data well. The parameter estimates and bootstrap results are detailed in Tables 3, 4 and 5 for each model.

**Table 3:** Final pharmacokinetic parameter estimates and bootstrap results for SB12 and reference eculizumab

**Parameter**	**Description (units)**	**Final model**			**Bootstrap**	
		**Estimate**	**RSE** **(%)**	**Shrinkage (%)**	**Median**	**95% CI**
**Fixed effect**						
CL = *θ*_1_ × (WT/80.9)^*θ*7^						
*θ*_1_	Clearance (L/h)	0.0174	0.91	-	0.0174	(0.0171, 0.0177)
*θ*_7_	Exponent to weight	1.1400	7.46	-	1.13	(0.95, 1.31)
*V*_*c*_ = *θ*_2_ × (WT/80.9)^*θ*8^						
*θ*_2_	Volume of central compartment for healthy subjects (L)	3.47	0.93	-	3.47	(3.40, 3.53)
*θ*_2__pat	Volume of central compartment for PNH patients (L)	5.68	6.11	-	5.68	(4.96, 6.39)
*θ*_8_	Exponent to weight	0.8630	10.57	-	0.8675	(0.67, 1.06)
*V*_*P*_	Volume of peripheral compartment (L)	0.79	3.28	-	0.80	(0.73, 0.85)
*Q*	Intercompartmental clearance between V_C_ and *V*_*P*_ (L/h)	0.0134	7.46	-	0.0135	(0.0100, 0.0150)
**Inter-individual variability**						
*ω*_CL_	Inter-individual variability for CL (%)	15.62	10.08	2.42	15.40	(13.90, 17.00)
*ω*_VC_	Inter-individual variability for V_C_ (%)	12.74	11.24	11.8	12.60	(11.30, 14.10)
*ω*_VP_	Inter-individual variability for V_P_ (%)	36.80	35.67	23.7	34.10	(22.90, 66.00)
*ρ*_CL−VC_	Correlation between CL and V_C_	0.54	16.79	-	0.54	(0.40, 0.65)
**Residual error**						
*σ*_prop_	Proportional error (%)	11.70	5.55	-	11.60	(10.40, 12.90)

*CI* confidence interval, *CL* clearance, *V*_*c*_ volume of central compartment, *V*_*p*_ volume of peripheral compartment, *Q* intercompartmental clearance, *RSE* relative standard error, *WT* weight

**Table 4:** Final pharmacodynamic parameter estimates for terminal complement activity and bootstrap results for SB12 and reference eculizumab

**Parameter**	**Description (units)**	**Final model**			**Bootstrap**	
		**Estimate**	**RSE (%)**	**Shrinkage (%)**	**Median**	**95% CI**
**Fixed effect**						
*E*0	Baseline terminal complement activity in healthy subjects (%)	85.90	1.12	-	85.90	(84.00, 87.90)
*E*0_pat	Baseline terminal complement activity in patients with PNH (%)	101.00	3.22	-	101.00	(94.45, 108.00)
*I*_max_	Maximum effect of inhibition in healthy subjects	0.93	0.34	-	0.93	(0.92, 0.94)
*I*_max__pat	Maximum effect of inhibition in patients	0.88	0.63	-	0.88	(0.86, 0.89)
*IC*_50_	Serum concentration achieving 50% of *I*_max_ (μg/mL)	36.60	1.57	-	36.60	(35.50, 37.80)
*H*	Hill coefficient	4.56	2.43	-	4.56	(4.35, 4.77)
**Inter-individual variability**						
*ω*_*E*0_	Inter-individual variability for *E*_0_ (%)	15.29	14.68	16.39	15.10	(12.80, 17.30)
*ω*_Imax_	Inter-individual variability for *I*_max_ (%)	2.65	11.09	14.48	2.60	(2.30, 2.90)
*ω*_IC50_	Inter-individual variability for *IC*_50_ (%)	22.74	19.37	8.75	22.20	(15.50, 26.70)
**Residual error**						
*σ*_prop_	Proportional error (%)	18.60	2.71	-	18.60	(-18.10, 19.60)

*CI* confidence interval, *PNH* paroxysmal nocturnal haemoglobinuria, *RSE* relative standard error

**Table 5:** Final pharmacokinetic-pharmacodynamic-efficacy parameter estimates and bootstrap results for SB12 and reference eculizumab

**Parameter**	**Description (units)**	**Final model**			**Bootstrap**	
		**Estimate**	**RSE (%)**	**Shrinkage (%)**	**Median**	**95% CI**
**Fixed effect**						
LL0	Baseline LDH in terminal complement activity vs. LDH relationship (U/L)	206.00	8.59	-	204.00	(110.30, 236.00)
LMAX	Maximum effect in terminal complement activity vs. LDH relationship (U/L)	1680.00	10.24	-	1670.00	(1310.00, 2100,00)
LC50	Terminal complement activity achieving 50% of LMAX (%)	39.00	7.13	-	39.20	(29.22, 51.06)
LGAM	Hill coefficient	4.30	18.91	-	4.39	(2.63, 7.70)
**Inter-individual variability**						
*ω*_LL0_	Inter-individual variability for LL0 (%)	28.97	51.74	29.3	27.80	(11.32, 74.43)
*ω*_LMAX_	Inter-individual variability for LMAX (%)	81.57	22.75	7.27	66.00	(48.50, 84.59)
*ω*_LGAM_	Inter-individual variability for LGAM (%)	55.91	33.60	12.00	52.80	(28.92, 80.07)
**Residual error**						
*σ*_prop_	Proportional error (%)	32.00	6.61	-	31.60	(27.42, 35.80)

*CI* confidence interval, *LDH* lactate dehydrogenase, *RSE* relative standard error

#### Comparison of pharmacokinetic, pharmacodynamic and efficacy parameters between SB12 and ECU

**Fig. 5:**
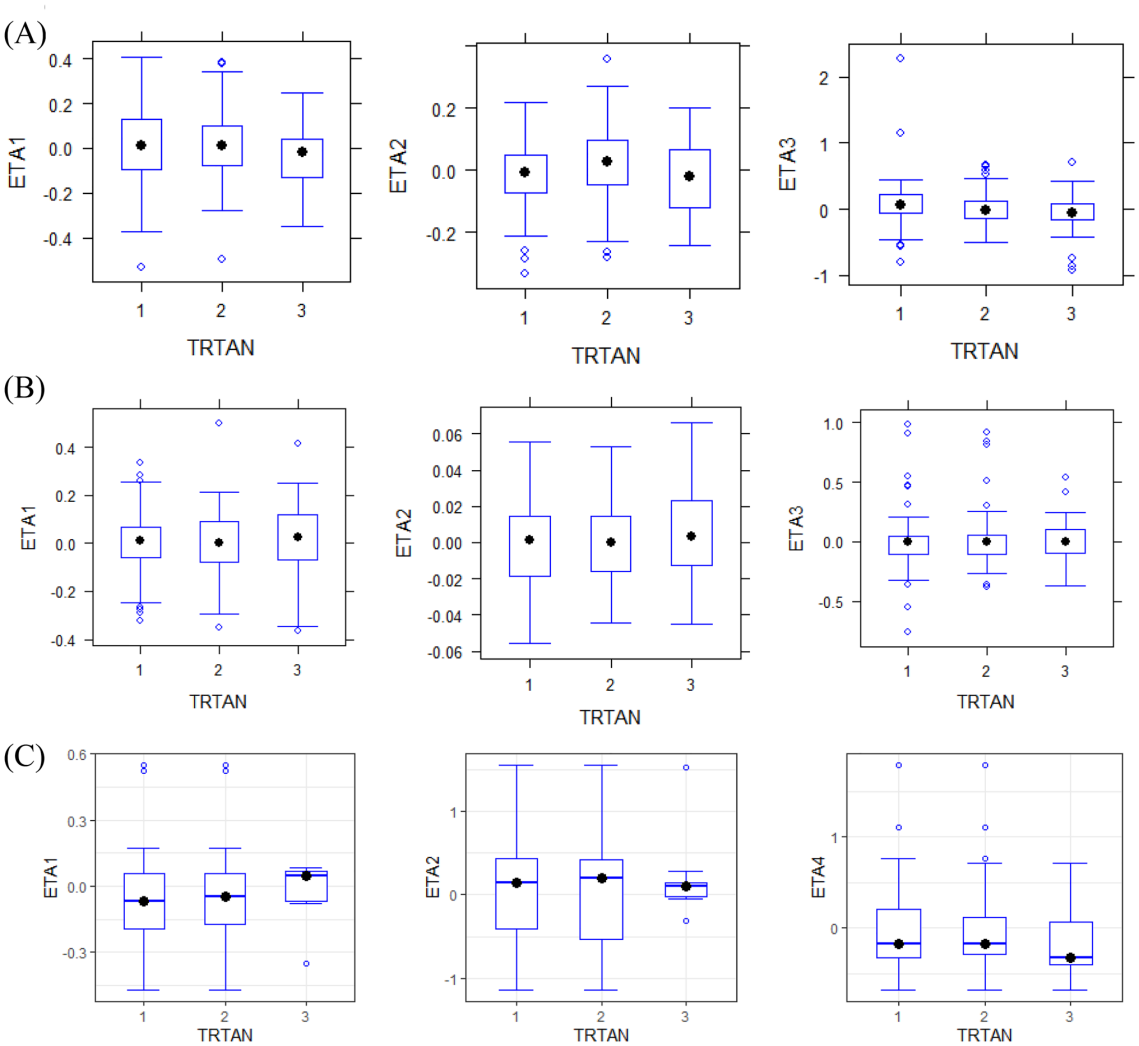
Boxplots of inter-individual variability stratified by treatment group in **A** pharmacokinetic model, ETA1, ETA2 and ETA3 indicate IIV of CL, *V*_*c*_ and *V*_*p*_, respectively; **B** pharmacodynamic model for terminal complement activity, ETA1, ETA2 and ETA3 indicate IIV of *E*_0_, *I*_max_ and *IC*_50_, respectively, and **C** pharmacokinetic-pharmacodynamic-efficacy model, ETA1, ETA2 and ETA4 indicate IIV of LL0, LMAX and LGAM, respectively. **A**–**C** TRTAN indicates treatment group; 1 = SB12, 2 = EU- ECU, 3 = US-ECU

Likelihood ratio test assessing the goodness of fit and visual inspection of boxplots was used to confirm the effect of the treatments, SB12 and ECU, on the ETAs of PK, PD and efficacy parameters. There are no statistically significant parameters for the inclusion of the treatment effect. The distribution profiles of ETAs of SB12, EU-ECU and US-ECU in boxplots were highly similar. It implied that the serum concentration–time profiles, terminal complement activity-time profiles and LDH-time profiles of SB12 and ECU could be explained by the same PK, PD and efficacy parameters. Also, no subgroup effect was identified between Asian vs. non-Asian and Chinese vs. non-Chinese, and they were similar. The boxplots of each ETA of PK, PD and efficacy parameters are presented by treatment group (Fig. 5) and by subgroup (Appendix Fig. A1).

## Discussion

The PK, PD and efficacy of SB12 and ECU were studied in a total of 240 healthy subjects treated with a single dose of 300-mg intravenous infusion, and 49 patients with PNH were given recommended dosing regimen of an initial dose of 600 mg weekly for the first 4 weeks followed by 900 mg for the fifth dose 1 week later, then 900 mg every 2 weeks thereafter.

The PK observations of SB12 and ECU from phase I and phase III studies were comparable to those observed in ECU from the published references, which enabled this modeling of pooled studies with phase I and phase III data without any correction or adjustments to the serum concentrations [10, 33]. Current data from two studies had a limitation in adequately capturing the difference in PKs between healthy subjects and PNH patients. The direct comparison between healthy subjects and PNH patients was not feasible because the condition for measuring drug concentration in blood such as dosing regimen, blood sampling points and achievement status of steady-state is very different. Therefore, it was necessary to use published references for supporting the differences of PKs between healthy subjects and PNH patients [10, 33]. The differences in pharmacokinetics between healthy subjects and PNH patients were reflected in the base PK model prior to the proper covariate analysis. A two-compartment model with a proportional residual model successfully described the observed PK data, and covariate analysis identified the significant effect of weight on *CL* and *V*_*c*_, which was in line with previous findings of ECU [10].

The PK/PD model for terminal complement activity and PK/efficacy model for LDH (not included) were separately built to evaluate the effect of serum concentration of the drug on each PD and efficacy markers separately, which may have better clinical utility and availability for the direct comparison to the reference values. In the analysis, the PD and efficacy markers were also linked in continuum to build the PK/PD/efficacy model, which allowed us to explore the relationship between terminal complement activity and LDH in patients.

The direct response model was used to link serum concentrations of the drug to terminal complement activity, which was well described by an inhibitory sigmoid *E*_max_ model. To describe LDH profiles in patients with PNH, the indirect response model inhibiting synthesis of terminal complement complex by serum concentrations was used (not included). To establish PD/efficacy models in continuum, the relationship between terminal complement activity and LDH was well described by a direct sigmoid *E*_max_ model. Other covariates potentially affecting the PD and efficacy parameters were a few laboratory parameters associated with kidney functions for terminal complement activity and red blood cells and liver functions for LDH, respectively. However, none of them were incorporated into the PD and efficacy models because physiological plausibility and evidence for the clinical significance of the identified relationships are lacking. In the PK/PD model, there was a difference between healthy subjects and PNH patients in *E*_0_ and *I*_max_, and there was no covariate selected for efficacy model in patients. As a result of PK/PD modeling and PK/PD/efficacy modeling, there were no significant differences in PK, PD and efficacy among treatment groups in both populations of healthy subjects and patients with PNH.

In previous study, the PK/PD relationship was assessed using serum haemolytic activity in patients with RA, IMG, PNH and systemic lupus erythematous (SLE). The concentration-dependent blockade of haemolytic activity showed an inverse relationship, and higher eculizumab concentration correlated with complete inhibition of complement activity [26, 34]. Complete inhibition of complement haemolytic activity was evaluated at eculizumab concentrations as low as 29–55 μg/mL and 11–35 μg/mL in RA and SLE patients. Also, the European Medicines Agency (EMA) scientific report using pooled data from various clinical studies including single and multiple-dose studies with patients suggested that a serum eculizumab concentration of 35 μg/mL is sufficient to completely inhibit complement activity. Therefore, the dosing schedule of 600 mg in the initiation phase (i.e. weekly for the first 4 weeks) followed by 900 mg for the fifth dose 1 week later, then 900 mg every 2 weeks was selected as the most optimal dosing regimen to achieve complete complement blockade in almost all PNH patients. The PK/PD model development in this study has some limitations. First, the effect of the relative range of sampled serum concentrations to *IC*_50_ and sigmoidicity between exposure (i.e. PK) and response (i.e. PD) on the parameter estimation performance using intensive sampling design was simulated in previous study [35]. PD parameters using sigmoid *E*_max_ model were accurately and precisely estimated when the *C*_max_ was more than 0.85 *IC*_50_ units [35]. In case of SB12 and ECU in single-dose phase I study with healthy subjects, the *C*_max_ value was more than 0.85 *IC*_50_ units; thus, the PD parameters estimated using inhibitory sigmoid *E*_max_ model could be judged to be accurate and precise. However, the PK/PD model using only *C*_trough_ or steady-state data in phase III study may not precisely estimate the PD parameter, so PD parameters from phase III study may be carefully interpreted [35]. It may be related to the high inter-individual variability of efficacy parameters estimated from PD/efficacy model. Second, the IPP method was used to sequentially link PK to PD and PK/PD to efficacy for this population modeling, and the parameter estimates were relatively stable, and prediction for median value was adequate. However, there is a chance that population PK prediction and data (PPP&D) or simultaneous (SIM) method or modelling in log scale may improve the efficacy model fit [28]. Third, nonlinear elimination term (i.e. Michaelis–Menten elimination) was not applied to the PK model. At the high concentration of non-therapeutic dose range, serum ECU concentration shows nonlinear PK, and PK model integrating a nonlinear elimination allowed a better prediction of ECU concentration than a linear model [37]. Although the nonlinear elimination term was not applied because most serum ECU concentrations in this study were within the therapeutic range, two-compartment model with both linear and Michaelis–Menten elimination may more adequately describe the ECU PK.

Asian and non-Asian patients appeared to have similar SB12 and ECU PK profiles, and there were no clinically meaningful differences with regard to terminal complement activity and LDH. Also, there were no differences in PK, PD and efficacy parameters between Chinese and non-Chinese. However, it should be noted that data from Chinese subjects were only available for eight patients (16.0%) of the phase III study. Nevertheless, based on the totality of the data, it would appear that SB12 and ECU dose adjustments and dosing regimen changes are not required in specific populations (e.g. Asian and Chinese patients).

Another complement C5-inhibitor, ravulizumab, has been approved for the treatment of PNH in the US and Europe and maintains a sustained inhibition of complement for 8 weeks following intravenous infusion. Population PK/PD/efficacy modelling and simulation of ravulizumab were performed and described by the manufacturer in the approval review documents of the US Food and Drug Administration and EMA [37, 38]. The PK of ravulizumab was well described with a linear two-compartment model, with weight at baseline as covariates on clearance and volume of distribution same as ECU. This analysis confirmed that the dosing regimen of phase III is the lowest dose with maximal effect, and the dosing regimen of ravulizumab was optimized by patient’s body weight. The PK/efficacy relationship was modelled using an indirect response PK/PD model using data from patients with PNH from phase II and III studies unlike population PK/PD/efficacy modelling of ECU. The indirect response model for PK/PD relationship assumed homeostasis of the response variable prior to drug treatment governed by a zero-order production and a first-order degradation process for the efficacy variable (i.e. serum LDH). In the indirect response model, the drug-induced serum LDH lowering effect was modelled by inhibiting production of response.

One strength of this population PK/PD/efficacy modelling of ECU is the relatively large sample size of patients, particularly considering that PNH is a rare disease. Additionally, the inclusion of healthy subjects enhances the comprehensiveness of the model, resulting in a robust description of the PK, PD and efficacy profiles along with their relationships.

In conclusion, this population modeling showed PK, PD and efficacy similarities between SB12 and ECU in pooled population of healthy subjects and PNH patients, supporting the totality of evidence on biosimilarity for SB12. In addition, results of this PK/PD/efficacy analysis provide evidence-based confirmation of the recommended dosing regimen for intravenous ECU in adult patients with PNH, which comprises 600 mg weekly for the first 4 weeks followed by 900 mg for the fifth dose 1 week later, then 900 mg every 2 weeks thereafter.

### Supplementary Information

Below is the link to the electronic supplementary material.

Supplementary file1 (DOCX 254 KB)

## Data Availability

Upon request, and subject to certain criteria, conditions and exceptions, Samsung Bioepis will provide access to individual de-identified participant data to researchers whose proposal meets the research criteria and other conditions and for which an exception does not apply. Proposals should be directed to the corresponding author. For access, data requestors must enter into a data access agreement with Samsung Bioepis.
