# Supplementary material for: Population pharmacokinetic, pharmacodynamic and efficacy modeling of SB12 (proposed eculizumab biosimilar) and reference eculizumab
Source: Eur J Clin Pharmacol. 2024 May 30;80(9):1325–38. doi: 10.1007/s00228-024-03703-8 (PMC11303580; doi:10.1007/s00228-024-03703-8)
Supplement: Supplementary file 1 — Supplementary file1 (DOCX 254 KB) [file 228_2024_3703_MOESM1_ESM.docx]

| **(A_1)** |  |  |
| --- | --- | --- |
| **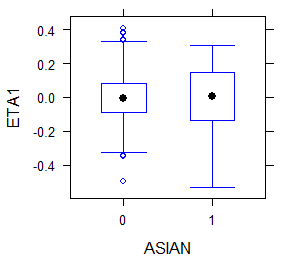** | **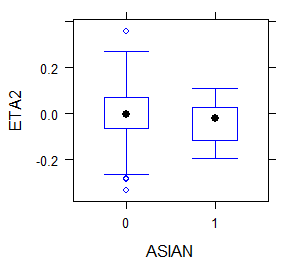** | **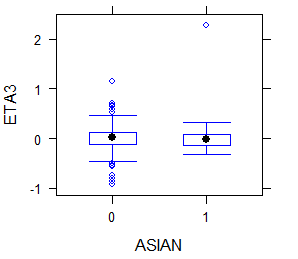** |
| **(A_2)** |  |  |
| **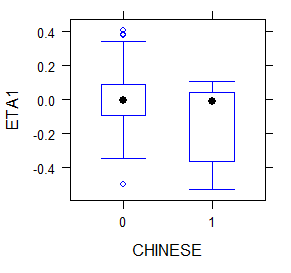** | **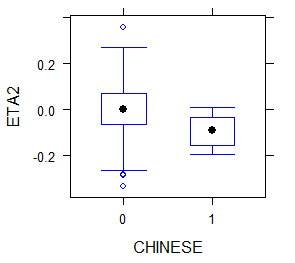** | **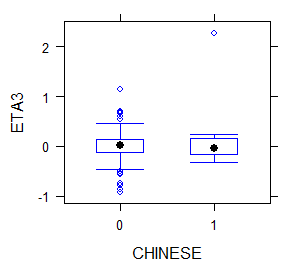** |
| **(B_1)** |  |  |
| **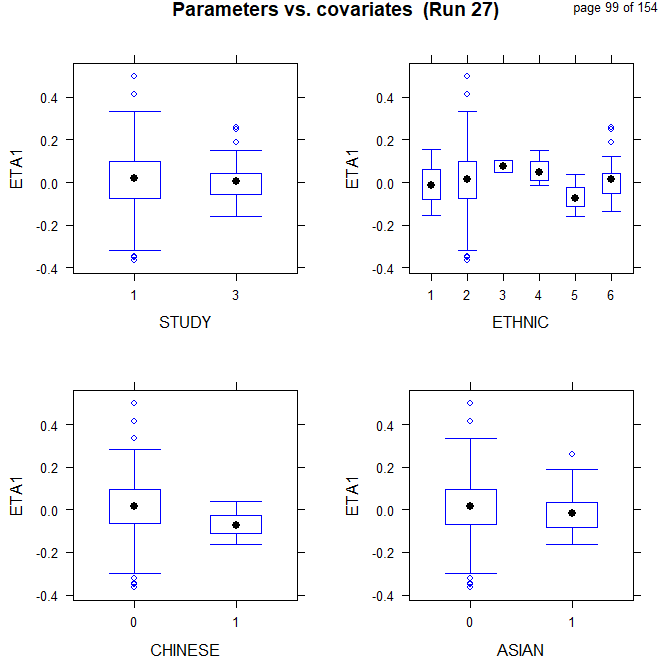** | **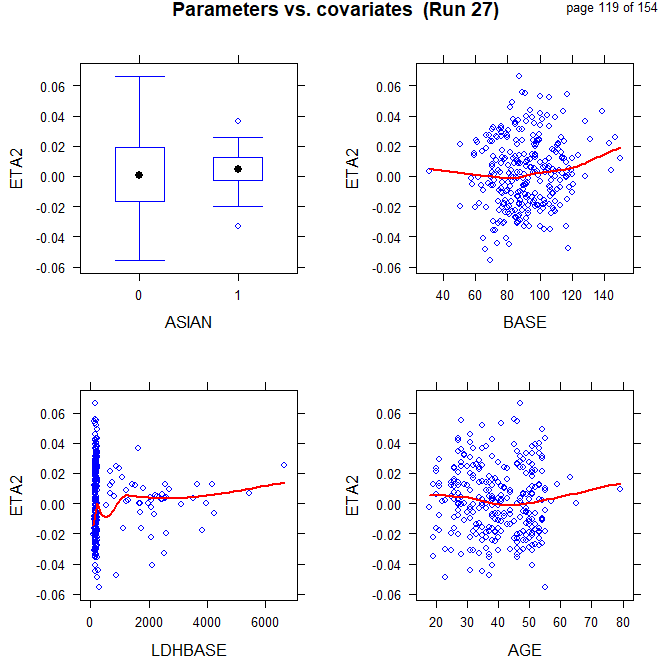** | **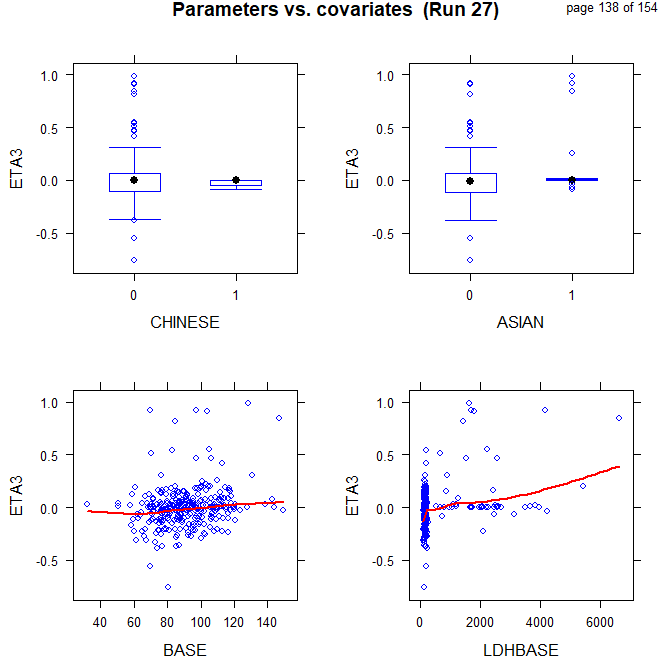** |
| **(B_2)** |  |  |
| **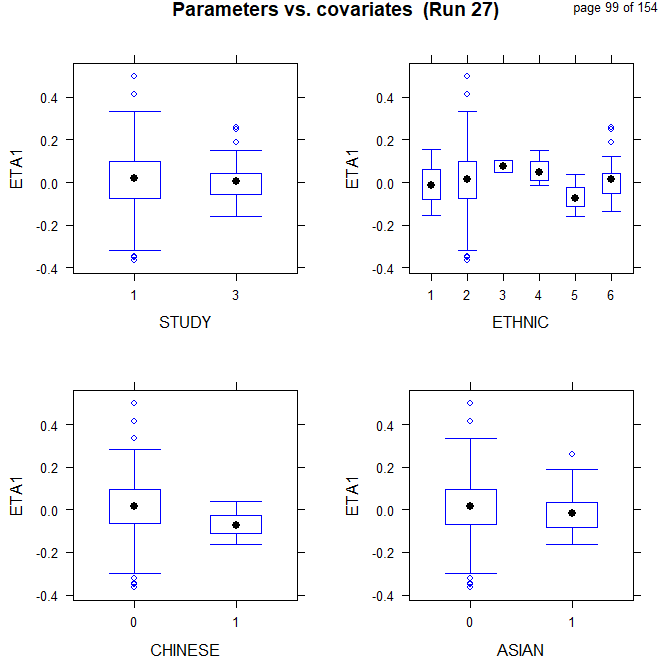** | **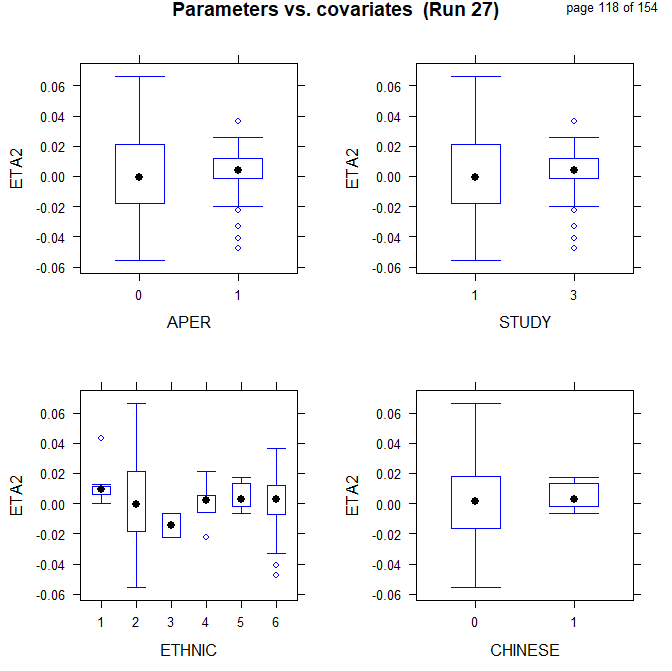** | **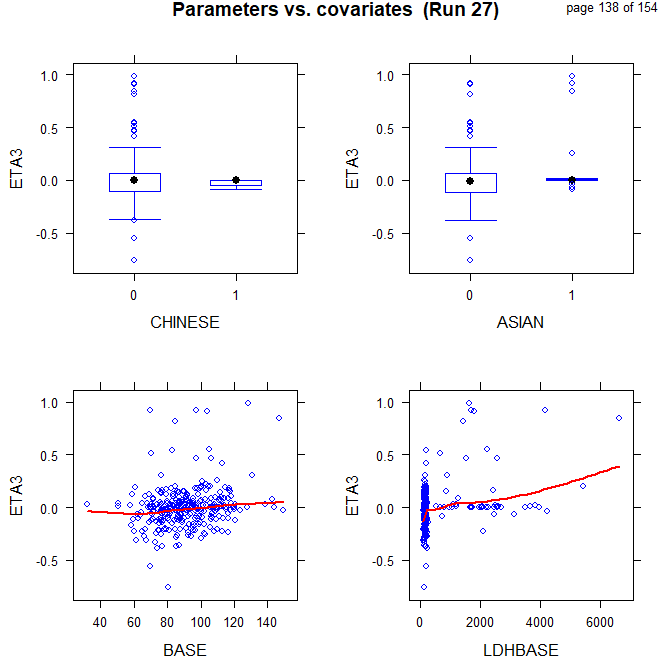** |
| **(C_1)** |  |  |
| **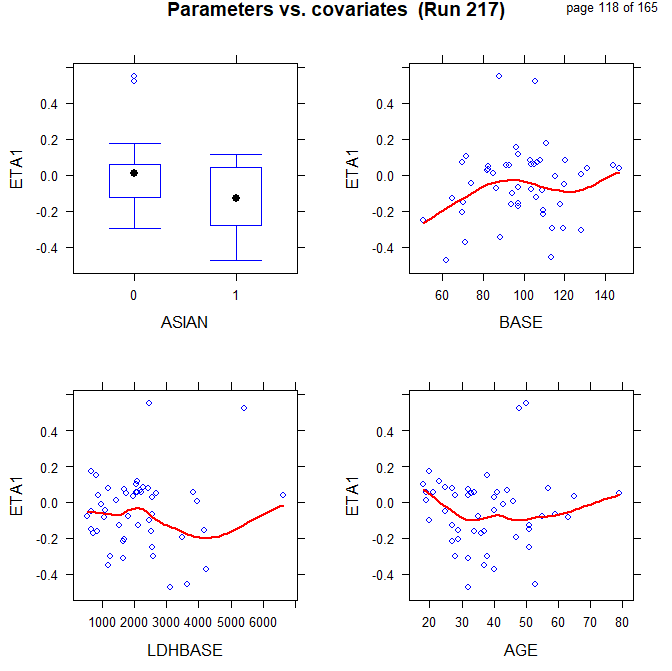** | **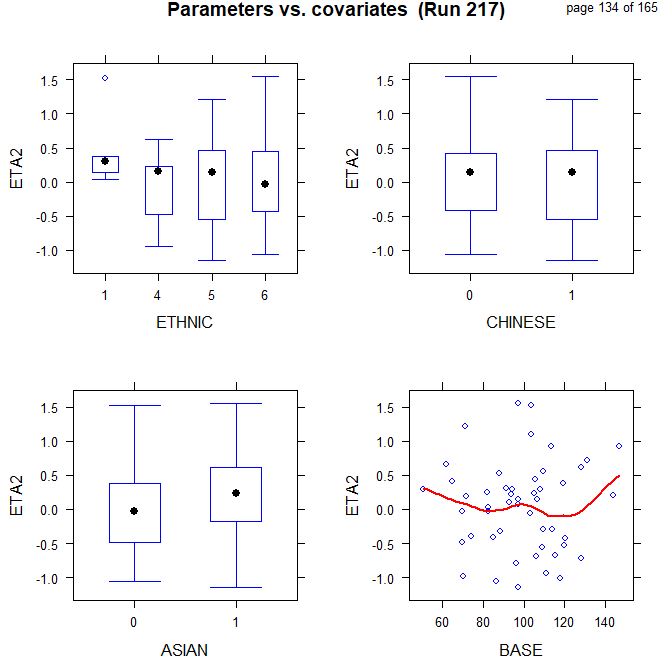** | **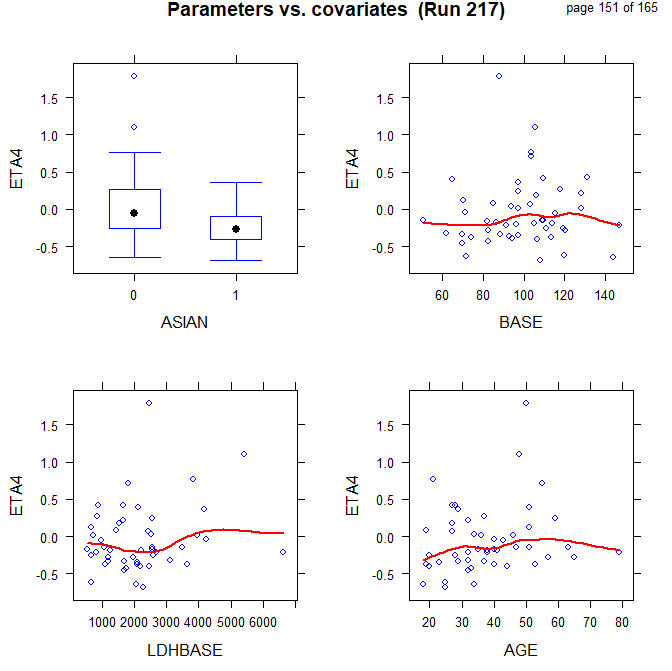** |
| **(C_2)** |  |  |
| **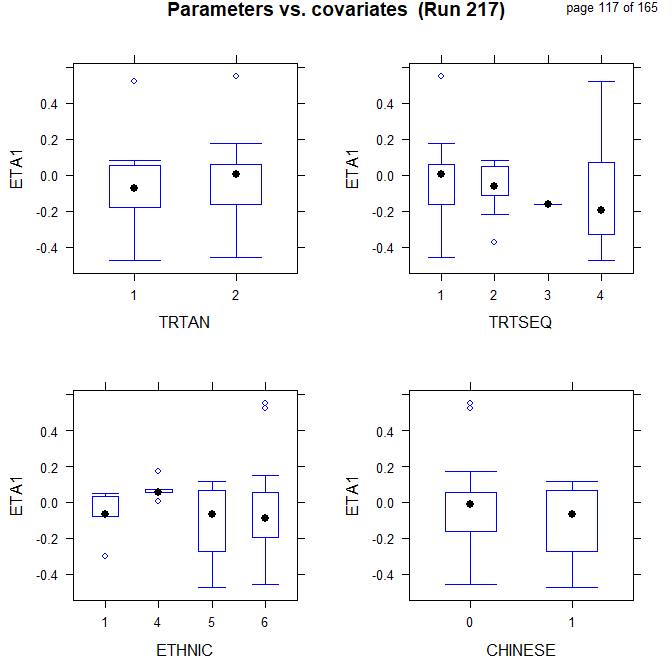** | **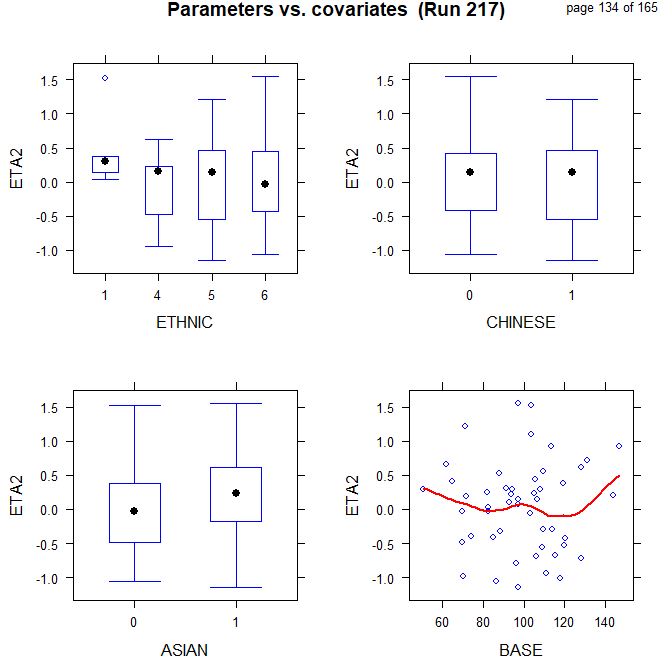** | **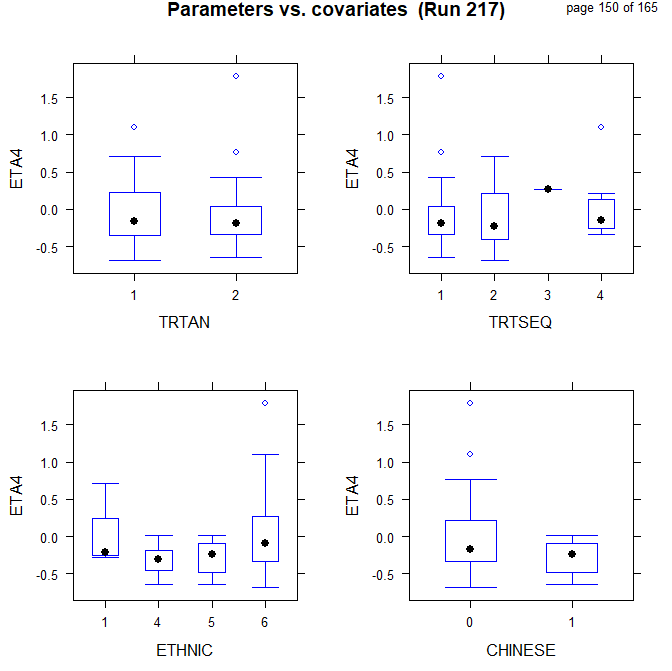** |

Figure A1. Boxplots of inter-individual variability stratified by Asian vs. Non-Asian and Chinese vs. Non-Chinese in (A) PK model, ETA1, ETA2 and ETA3 indicates IIV of CL, Vc, and Vp, respectively, (B) PD model for terminal complement activity, ETA1, ETA2 and ETA3 indicates IIV of E0, Imax, and IC50, respectively, (C) PK-PD-Efficacy model, ETA1, ETA2, and ETA4 indicates IIV of LL0, LMAX, and LGAM respectively. (A_1) - (C_1) ASIAN indicates; 1 = Asian, 0 = Non-Asian,
(A_2) - (C_2) CHINESE indicates; 1 = Chinese, 0 = Non-Chinese

Table A1. Final pharmacokinetic parameter estimates and bootstrap results for SB12 and ECU in healthy subjects

| Parameter | Description (units) | Final model | | | Bootstrap | | |
| --- | --- | --- | --- | --- | --- | --- | --- |
|  |  | Estimate | RSE  (%) | Shrinkage (%) | Median | 95% CI | |
| Fixed effect | | | | | | | |
| CL = θ_1_ × (WT/82.4)^θ7^ | | | | | | | |
| θ_1_ | Clearance (L/h) | 0.0177 | 0.89 |  | 0.0177 | | (0.0174, 0.0180) |
| θ_7_ | Exponent to weight | 0.7630 | 18.35 |  | 0.766 | | (0.542, 1.01) |
| V_c_ = θ_2_ × (WT/82.4)^θ8^ | | | | | | | |
| θ_2_ | Volume of central compartment (L) | 3.50 | 0.91 |  | 3.49 | | (3.43, 3.56) |
| θ_8_ | Exponent to weight | 0.6840 | 16.37 |  | 0.6850 | | (0.4920, 0.8670) |
| V_p_ | Volume of peripheral compartment (L) | 0.7850 | 3.41 |  | 0.7840 | | (0.7320, 0.8380) |
| Q | Intercompartmental clearance between V_C_ and V_P_ (L/h) | 0.0136 | 7.43 |  | 0.0136 | | (0.0117, 0.0155) |
| Inter-individual variability | | | | | | | |
| ω_CL_ | Inter-individual variability for CL (%) | 13.55 | 10.33 | 1.54 | 13.4 | | (12.1, 14.8) |
| ω_Vc_ | Inter-individual variability for V_C_ (%) | 12.66 | 9.62 | 3.95 | 12.5 | | (11.4, 13.7) |
| ω_Vp_ | Inter-individual variability for V_P_ (%) | 31.76 | 20.81 | 15.28 | 30.7 | | (24.2, 37.4) |
| ρ_CL−VC_ | Correlation between CL and V_C_ | 0.553 | - |  | 0.554 | | (0.461, 0.654) |
| Residual error | | | | | | | |
| σ_prop_ | Proportional error (%) | 8.49 | 3.93 |  | 8.48 | | (7.82, 9.13) |

CI: confidence interval; CL: clearance; Q: intercompartmental clearance; RSE: relative standard error; Vc: volume of central compartment; Vp: volume of peripheral compartment; WT: weight (kg)

Table A2. Final pharmacodynamic parameter estimates and bootstrap results for SB12 and ECU in healthy subjects

| Parameter | Description (units) | Final model | | | Bootstrap | | |
| --- | --- | --- | --- | --- | --- | --- | --- |
|  |  | Estimate | RSE  (%) | Shrinkage (%) | Median | 95% CI | |
| Fixed effect | | | | | | | |
| E0 | Baseline terminal complement activity (%) | 86.20 | 1.11 |  | 86.20 | | (84.30, 88.00) |
| I_max_ | Maximum effect of inhibition | 0.93 | 0.34 |  | 0.931 | | (0.925, 0.938) |
| IC_50_ = θ_3_ × (Baseline/87.75)^θ8^ | | | | | | | |
| θ_3_ | Serum concentration achieving 50% of I_max_ (ug/mL) | 36.20 | 1.09 |  | 36.20 | | (35.40, 36.90) |
| θ_8_ | Effect of baseline terminal complement activity on IC_50_ | 0.14 | 38.79 |  | 0.14 | | (0.02, 0.25) |
| H | Hill coefficient | 4.52 | 2.41 |  | 4.53 | | (4.31, 4.52) |
| Inter-individual variability | | | | | | | |
| ω_E0_ | Inter-individual variability for E0 (%) | 15.88 | 15.22 | 12.84 | 15.7 | | (13.3, 18.1) |
| ω_Imax_ | Inter-individual variability for I_max_ (%) | 2.63 | 11.68 | 11.74 | 2.60 | | (2.30, 2.90) |
| ω_IC50_ | Inter-individual variability for IC50 (%) | 13.33 | 14.77 | 9.08 | 13.2 | | (11.3, 15.1) |
| Residual error | | | | | | | |
| σ_prop_ | Proportional error (%) | 18.00 | 2.59 |  | 18.30 | | (17.50, 19.30) |

Baseline: baseline of terminal complement activity (%); CI: confidence interval; IC_50_: serum concentration achieving 50% of I_max_ (ug/mL); RSE: relative standard error
